# CBL-Interacting Protein Kinase OsCIPK18 Regulates the Response of Ammonium Toxicity in Rice Roots

**DOI:** 10.3389/fpls.2022.863283

**Published:** 2022-04-29

**Authors:** Tong Sun, Ting Wang, Yalin Qiang, Gangqing Zhao, Jian Yang, Hua Zhong, Xiaojue Peng, Jing Yang, Yangsheng Li

**Affiliations:** ^1^State Key Laboratory of Hybrid Rice, College of Life Sciences, Wuhan University, Wuhan, China; ^2^Department of Chemistry, University of Kentucky, Lexington, KY, United States; ^3^College of Life Sciences, Nanchang University, Nanchang, China

**Keywords:** ammonium toxicity, NH^+^_4_, OsCIPK18, rice (*Oryza sativa* L.), RNA-Seq

## Abstract

Ammonium (${\text{NH}}_{4}^{+}$) is one of the major nitrogen sources for plants. However, excessive ammonium can cause serious harm to the growth and development of plants, i.e., ammonium toxicity. The primary regulatory mechanisms behind ammonium toxicity are still poorly characterized. In this study, we showed that OsCIPK18, a CBL-interacting protein kinase, plays an important role in response to ammonium toxicity by comparative analysis of the physiological and whole transcriptome of the T-DNA insertion mutant (*cipk18*) and the wild-type (WT). Root biomass and length of *cipk18* are less inhibited by excess ${\text{NH}}_{4}^{+}$ compared with WT, indicating increased resistance to ammonium toxicity. Transcriptome analysis reveals that OsCIPK18 affects the ${\text{NH}}_{4}^{+}$ uptake by regulating the expression of OsAMT1;2 and other ${\text{NH}}_{4}^{+}$ transporters, but does not affect ammonium assimilation. Differentially expressed genes induced by excess ${\text{NH}}_{4}^{+}$ in WT and *cipk18* were associated with functions, such as ion transport, metabolism, cell wall formation, and phytohormones signaling, suggesting a fundamental role for OsCIPK18 in ammonium toxicity. We further identified a transcriptional regulatory network downstream of OsCIPK18 under ${\text{NH}}_{4}^{+}$ stress that is centered on several core transcription factors. Moreover, OsCIPK18 might function as a transmitter in the auxin and abscisic acid (ABA) signaling pathways affected by excess ammonium. These data allowed us to define an OsCIPK18-regulated/dependent transcriptomic network for the response of ammonium toxicity and provide new insights into the mechanisms underlying ammonium toxicity.

## Introduction

Nitrogen is one of the essential elements for plant growth and development, and ammonium (${\text{NH}}_{4}^{+}$) is the predominant nitrogen source for plants (Forde, 2002; Hirano et al., 2008; Li et al., 2014). When ammonium is supplied at an appropriate concentration, it promotes plant growth and development. However, the elevated concentration of ammonium can give rise to symptoms of ammonium toxicity in many plants (Britto and Kronzucker, 2002; Liu and Wirén, 2017). Ammonium toxicity-related phenotypes include stunted growth, short and thick roots, lack of root gravitropism, yellowing of leaves, and even plant death in severe cases (Esteban et al., 2016). In agricultural production, overaccumulation of ${\text{NH}}_{4}^{+}$ in the soil normally happened at the excessive application of nitrogen fertilizers and unreasonable fertilization methods, which in turn affects plant growth and reproduction, and seriously harms the yield of crops (Dave and Nilsson, 2005; Romano and Zeng, 2007). Ammonium toxicity is of great ecological and economic importance. It is meaningful to study the response mechanism of ammonium toxicity.

To regulate the stress from ammonium toxicity, plants need to balance the activities of uptake, production, and consumption of ${\text{NH}}_{4}^{+}$. Thus, the mechanisms of ammonium toxicity have been found to be related to several cellular phenomena or activities, including ion transport, rhizosphere acidification, photosynthesis, phytohormones, and ${\text{NH}}_{4}^{+}$ futile cycle (Zhu et al., 2009; Esteban et al., 2016; Alencar et al., 2019; Meier et al., 2020). For example, members of the ammonium transporter 1 (AMT1) subfamily play a major role in ${\text{NH}}_{4}^{+}$ uptake, including OsAMT1;1, OsAMT1;2, and OsAMT1;3, and simultaneous knockout of these three genes resulted in a 95% reduction in ${\text{NH}}_{4}^{+}$ uptake (Yutaka et al., 2003; Miller and Cramer, 2005; Konishi and Ma, 2021). Besides, other proteins, such as the potassium transporters and plasma membrane H^+^-ATPase, have also been reported to be involved in ${\text{NH}}_{4}^{+}$ transport (Kronzucker et al., 2001; Zhang M. et al., 2021). Meanwhile, ${\text{NH}}_{4}^{+}$ could be converted to organic nitrogen by assimilation through a metabolic cycle consisting of glutamine synthetase (GS) and glutamine-2-oxoglutarate aminotransferase (GOGAT) (Miflin and Habash, 2002; Li et al., 2014). Additionally, as the major players in the establishment and interconnection of plant signaling networks, phytohormones are also directly involved in ammonium toxicity (Krouk et al., 2010; Meier et al., 2020). For instance, the distribution of auxin is associated with ${\text{NH}}_{4}^{+}$-induced loss of root gravitropism (Zou et al., 2012). It has been proved that in rice the endogenous abscisic acid (ABA) could reduce reactive oxygen species (ROS) and free ${\text{NH}}_{4}^{+}$ of ammonium toxicity by regulating the SAPK9-bZIP20 pathway (Li et al., 2012; Sun et al., 2020). In spite of numerous studies on the mechanism of ammonium toxicity, the regulatory network related to ammonium toxicity in rice is still underdeveloped because of its complexity.

The calcineurin B-like protein (CBL), CBL-interacting protein kinase (CIPK) network, has been repetitively reported regulating several abiotic stress-induced signaling pathways, such as aluminum stress, K^+^, ${\text{NH}}_{4}^{+}$, ${\text{NO}}_{3}^{-}$ status, pH, salt stress, and oxidative stress (Hu et al., 2009; Yong et al., 2010; Hashimoto and Kudla, 2011). For example, in *Arabidopsis*, the AtCBL4-AtCIPK24 complex regulates the expression of the downstream functional gene AtSOS1 (Na^+^/H^+^ reverse transporter protein) to improve salt tolerance in roots (Sánchez-Barrena et al., 2005). AtCBL1/AtCBL9 interacts with AtCIPK23 to activate AtAKT1, a K^+^ channel protein localized at the plasma membrane, thereby regulating K^+^ uptake under low-K^+^ conditions (Xu et al., 2006). An increasing number of studies have reported that several CIPKs altered their transcript levels and phosphorylation status during the ammonium response to its toxicity. When exposed to excess ${\text{NH}}_{4}^{+}$, the AtCBL1-AtCIPK23 complex phosphorylates AtAMT1s to inhibit ${\text{NH}}_{4}^{+}$ transport in *Arabidopsis* (Straub et al., 2017). In rice, the expression of OsCIPK23, OsCIPK8, OsCIPK9, and OsCIPK14/15 was sensitive to exogenous ${\text{NH}}_{4}^{+}$ (Xuan et al., 2019). Among them, OsCIPK9 regulates ${\text{NH}}_{4}^{+}$-dependent root growth downstream of OsIDD10 (Xuan et al., 2019). However, research in this area is still poorly understood and needs to be further explored.

In this study, we used the T-DNA insertion mutant *cipk18* to investigate the role of OsCIPK18 in ammonium toxicity. By observing the physiological and biochemical difference between WT and the mutant *cipk18* in the absence and presence of excess ${\text{NH}}_{4}^{+}$, we found that *cipk18* exhibited the decreased toxicity of ammonium and ${\text{NH}}_{4}^{+}$ accumulation, whereas GS/GOGAT enzyme activity for ${\text{NH}}_{4}^{+}$ assimilation remained the same. It was further demonstrated that OsCIPK18 regulates free ${\text{NH}}_{4}^{+}$ in roots by affecting the expression of ${\text{NH}}_{4}^{+}$ transporters, including OsAMT1;2. Finally, RNA-seq was used to analyze the transcriptome data to further characterize the molecular mechanisms and identify promising candidates of transcription factors (TFs) affecting ammonium toxicity that may depend on the OsCIPK18 regulatory pathway.

## Materials and Methods

### Plant Materials and Growth Conditions

Wild-type lines of rice (*Japonica*, Hwayoung), T-DNA insertion mutant *cipk18*, and three complementary strains of *cipk18* (com1, com2, com3) were used in this study. The OsCIPK18 T-DNA insertion line (1C-05857) in the Hwayoung background was obtained from Kyung Hee University, Korea (Jeon et al., 2000). To generate the complemented lines, the 35S::OsCIPK18 fragment was constructed and transformed into *Agrobacterium tumefaciens* strain EHA105. Calli from *cipk18* were transformed as described by Cheng et al. (1998). Seeds used in this study were surface-sterilized with 1% sodium hypochlorite for 10 min, washed extensively with distilled water, and then incubated at 37°C in the dark for 2 days to break dormancy. Consistently growing germinating seeds were, respectively, transferred to two groups of hydroponic media for growth, namely, control (pure water) and ammonium toxicity treatment (4 mM NH_4_Cl solution) with no other nutrients present in the hydroponic media. All seedlings subjected to hydroponic treatments were grown in a temperature-controlled incubator set at 28°C, 14 h light/22°C, 10 h dark. All mature plants were grown and harvested in Wuhan, Hubei, and Lingshui, Hainan, China.

### Phenotyping of Rice Seedlings

The roots of seedlings of WT, *cipk18*, com1, com2, and com3 cultured for 3 days under ddH_2_O and 4 mM NH_4_Cl, respectively, were photographed at high resolution with a Nikon D7100 digital SLR camera, and root length data were obtained using smartRoot in ImageJ (Lobet and Draye, 2011). Seedlings of WT and *cipk18* that had been cultured for 7 days under control and NH_4_Cl treatments were selected and divided into groups of five plants to measure biomass, and the average value of individual plants was calculated and repeated three times. The roots of seedlings were drained and weighed directly to obtain fresh weight data, dried in an oven at 70°C for 3 days until their constant weight was obtained, and then weighed again to obtain dry weight data. The roots of 7-day-old rice seedlings were spread as far as possible, and high-resolution photographs were taken with a Nikon D7100 digital SLR camera to obtain root length, diameter, and number data using smartRoot in ImageJ.

### Tissue Ammonium Concentration Determination

The roots of 7-day-old rice seedlings were removed from the culture medium, rinsed three times with water, dried with paper towels to absorb the water, ground to powder with liquid nitrogen, weighed 0.1 g of the powder, and treated with 1 ml of 10 mM formic acid solution. After mixing thoroughly, they were centrifuged at 10,000 × g at 4°C for 10 min. Then, 1 ml OPA (100 mM KH_2_PO_4_, 100 mM K_2_HPO_4_, 3.75 mM *o*-phthalaldehyde, 2 mM β-ME) was added to 250 μl supernatants and reacted in the dark for 30 min, the absorbance was measured at 410 nm using infinite M200 Enzyme Scale. A standard curve was plotted with different concentrations of NH_4_Cl solution, and then it was used to calculate the concentration of tissue ${\text{NH}}_{4}^{+}$.

### Assays of GS and GOGAT Activity

The roots of WT and *cipk18* seedlings cultured for 7 days under two treatments described above were ground to powder with liquid nitrogen. Then, 0.1 g of tissue was used to measure GS and GOGAT activity according to the method described on the kit (cominbio). Absorbance value measurement was done using infinite M200 Enzyme Scale.

### RNA Extraction and Quantitative Real-Time Reverse Transcriptase-Polymerase Chain Reaction (RT-PCR)

To verify the expression changes of genes in WT and *cipk18* under ammonium toxicity, we extracted total RNA from roots of rice seedlings under ddH_2_O and 4 mM NH_4_Cl treatment and performed qRT-PCR using specific primers. RNA was extracted as one biological replicate from the roots of approximately 5–8 seedlings grown for 7 days in the same growth state under both treatments. We rapidly ground the treated roots in liquid nitrogen, extracted total RNA from rice samples using TRIzol (Invitrogen), removed genomic DNA from total RNA using DNase I (Thermo Scientific), and performed cDNA first-strand synthesis using M-MLV reverse transcriptase (Promega). qRT-PCR was done using 2× ChamQ SYBR qPCR Master Mix (UE), specific primers P+/P–, and cDNA using the Bio-Rad CFX96TM Real-Time System. The relative expressions were calculated using the 2^−ΔΔCT^ method. Three technical replicates were used to calculate the mean of the expression levels for each biological replicate, and three biological replicates were used to generate the mean of the expression levels for each sample. Graphing and significance analysis were performed using GraphPad Prism 6.

### RNA Sequencing and Data Analysis

The roots of WT and *cipk18* seedlings treated for 7 days under two treatments as described previously were divided into three replicates to prepare specific RNA-seq libraries, respectively, for a total of 12 libraries. The libraries were submitted to BGI for sequencing using the Illumina HiSeq 2000 platform. The raw data were filtered using trimmomatic (Bolger et al., 2014) to remove the low-quality reads, and the resulting high-quality reads were aligned to the NIP reference genome (ftp://ftp.ensemblgenomes.org/pub/plants/release-44/fasta/oryza_sativa/dna/) using STAR software (Dobin et al., 2013). After alignment, the raw counts were normalized to trimmed mean of M value (TMM) using RSEM (Li and Dewey, 2011). Difference analysis between the two samples was performed using DESeq2, where genes with *p*_adj_ < 0.05 and |log2FC| > 1 were identified as differential genes. Gene Ontology (GO) enrichment analysis was performed using TBtools, and the GO background was provided on the AgriGO V2.0 website (Tian et al., 2017; Chen et al., 2020). Kyoto Encyclopedia of Genes and Genomes (KEGG) analysis was done using the R package from clusterprofiler (Yu et al., 2012).

### TF Prediction

Transcription factor prediction was performed on the plantregmap website (http://plantregmap.gao-lab.org/), and the predicted results were filtered. TF network visualization was done by Cytoscape.

### Statistical Analysis

All statistical analyses were conducted using GraphPad Prism 6. The error bars in all the charts represent the standard deviation of the mean. The different letters above the error bar represent significant differences between groups. The comparison method uses one-way ANOVA combined with Duncan's *post-hoc* multiple test method. *p* < 0.05 was set as the significance cutoff.

## Results

### Knockdown of OsCIPK18 Enhances Ammonium Resistance in Rice Roots

**Figure 1:**
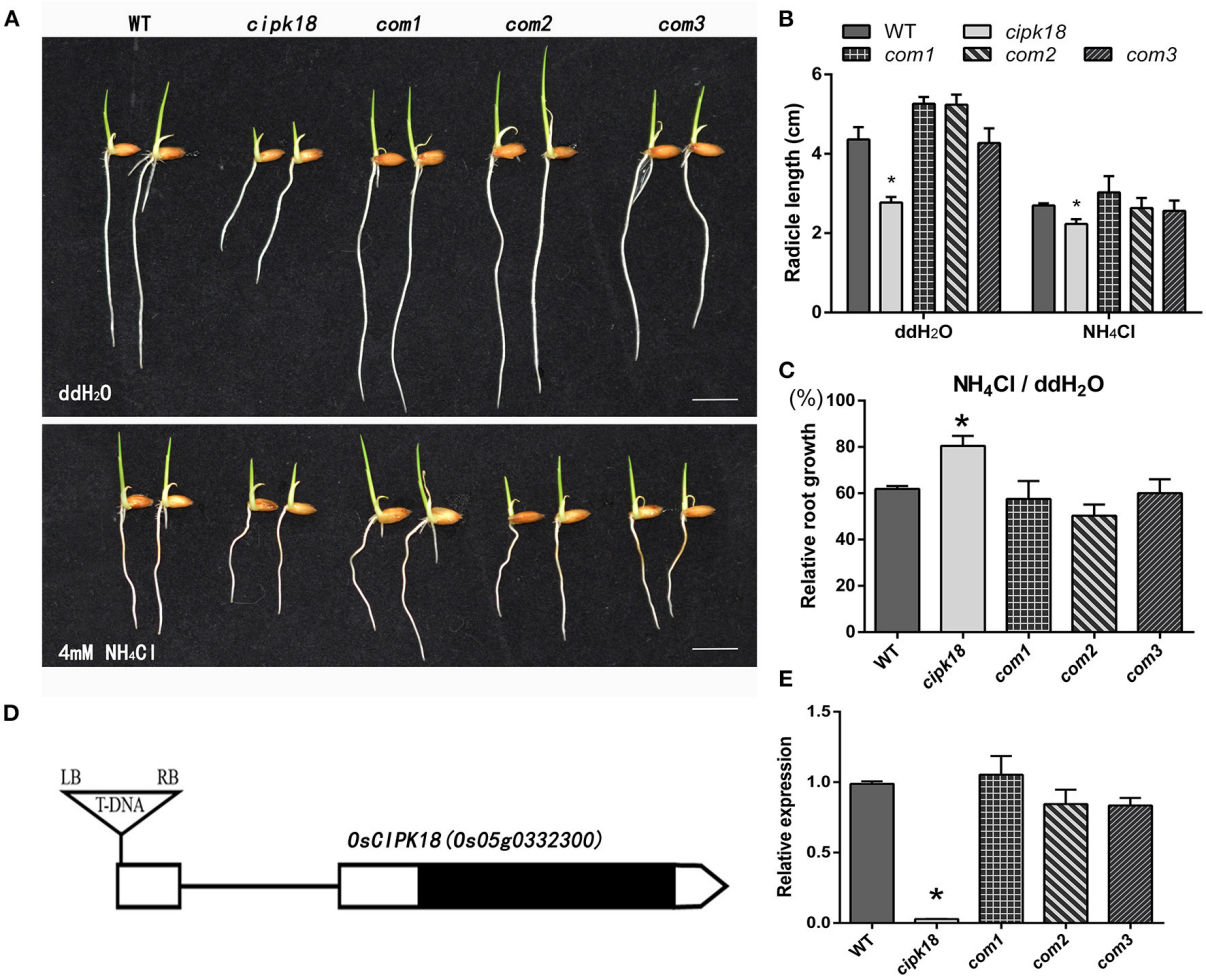
The mutant *cipk18* shows low levels of ammonium toxicity. **(A,B)** Rice seedlings phenotypes **(A)**, radicle length **(B)** of 3-day-old WT, *cipk18*, and three complementary materials of *cipk18* (com1, com2, com3) under treatments with ddH_2_O and 4 mM NH_4_Cl, respectively. Scale bar = 1 cm. **(C)** Compared with the control, relative root growth of the five lines under 4 mM NH_4_Cl. **(D)** Gene structures of the OsCIPK18 insertion mutant. UTRs are shown as white boxes and exons as black box. Triangle indicates T-DNA insertion sites. **(E)** Relative expression of OsCIPK18 in WT, *cipk18*, and three complementary materials of *cipk18*. Data are means ± SDs, significant differences using Student's *t*-test: *p < 0.05. RB, right border of T-DNA; LB, left border of T-DNA.

To screen the specific CIPK associated with ammonium toxicity in rice, we compared the ammonium resistance of WT with the mutant of OsCIPK T-DNA insertion. Ammonium resistance was quantified based on the phenotype of the relative root growth (i.e., root growth in the presence of high ${\text{NH}}_{4}^{+}$ compared with root growth in water as control). Growth inhibition but relatively enhanced ammonium resistance was identified in a mutant (1C-05857), in which ammonium toxicity only shortened its root length by about 20%, but by about 40% in WT (Figures 1A–C). This mutant carried a homozygous T-DNA insertion in the 5′ untranslated region (UTR) of OsCIPK18 (*Os05g0332300*), leading to a knockdown of the OsCIPK18 transcript (Figures 1D,E, Supplementary Figure 1). We obtained three OsCIPK18 complementation lines (com1, com2, and com3) and confirmed that the increased ammonium resistance was attributed by the mutation of OsCIPK18 (Figures 1A–C,E). With increasing incubation time, *cipk18* consistently showed resistance to excess ${\text{NH}}_{4}^{+}$ in terms of fresh weight and root length compared to WT and complementary lines (Supplementary Figure 2).

**Figure 2:**
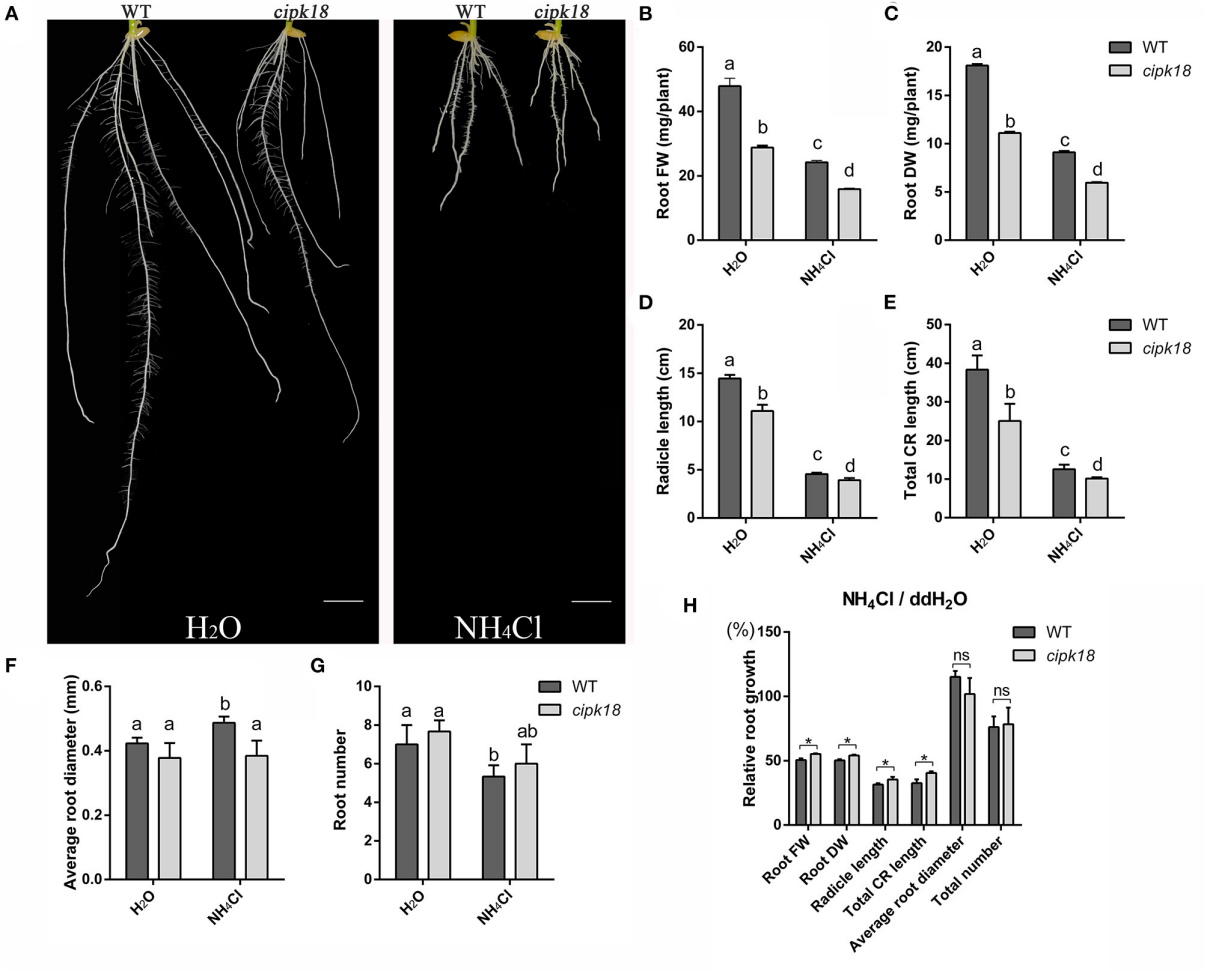
OsCIPK18 affects the root growth of rice seedlings under ammonium toxicity. Rice seedlings phenotypes **(A)**, root FW **(B)**, root DW **(C)**, radicle length **(D)**, total CR length **(E)**, average root diameter **(F)**, and root number **(G)** of 7-day-old WT and *cipk18* plant cultured in ddH_2_O and 4 mM NH_4_Cl, respectively. Scale bar = 1 cm. Different letters represent the significant difference, *p* < 0.05. **(H)** Relative root growth, i.e., the ratio of root growth data in the presence of ammonium toxicity to that of the control. Data are means ± SDs, significant differences using Student's *t*-test: *p < 0.05. FW, fresh weight; DW, dry weight; CR, crown root.

Furthermore, we investigated the development of root system between WT and the mutant *cipk18* under control and ammonium stress conditions. Exposing to excess ${\text{NH}}_{4}^{+}$, WT exhibited a significant decrease in biomass, root length and root number, and an increase in root diameter, which indicated that external high ${\text{NH}}_{4}^{+}$ severely affects rice root development of WT (Figures 2A–H). However, ammonium toxicity did not cause the same changes in root diameter and root number in the mutant *cipk18*. The effects of ammonium toxicity on root length of *cipk18* were diminished, and the fresh and dry weight also showed stronger ammonium resistance compared with WT (Figures 2A–H). Together, these results suggest that *cipk18* was more resilient to ammonium toxicity than WT, and protein kinase OsCIPK18 might be involved in the adjustment of the root structure of rice seedlings by ammonium toxicity.

### OsCIPK18 Affects the Accumulation of ${\text{NH}}_{4}^{+}$ Under Ammonium Toxicity Without Affecting Assimilation

**Figure 3:**
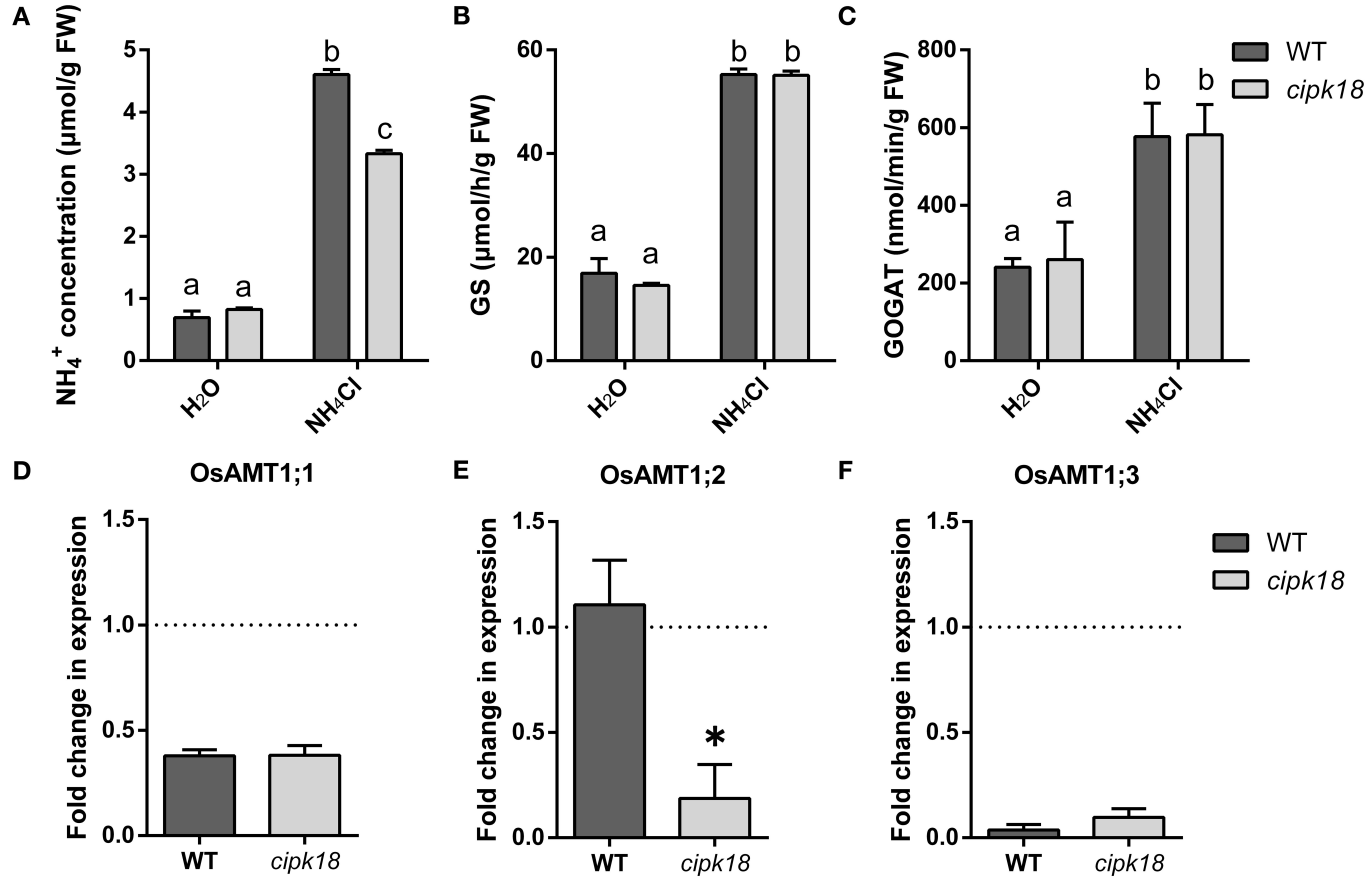
OsCIPK18 affects ${\text{NH}}_{4}^{+}$ accumulation in rice seedlings root under ammonium toxicity. ${\text{NH}}_{4}^{+}$ concentration **(A)**, GS activity **(B)**, and GOGAT activity **(C)** of 7-day-old rice seedlings root. Fold changes in the expression of OsAMT1;1 **(D)**, OsAMT1;2 **(E)**, and OsAMT1;3 **(F)** under ammonium toxicity compared with control, as determined by quantitative real-time PCR. Data are means ± SDs, different letters represent the significant difference, *p* < 0.05. GS, glutamine synthetase; GOGAT, glutamate synthase.

The excessive accumulation and assimilation of ${\text{NH}}_{4}^{+}$ in plants are considered as the important causes accounting for ammonium toxicity (Chen et al., 2013). To estimate the accumulation and assimilation of ${\text{NH}}_{4}^{+}$ in the roots of both WT and *cipk18*, we examined ${\text{NH}}_{4}^{+}$ content and enzymatic activity of GS/GOGAT under the control and ammonium stress conditions (Figures 3A–C). When only water was provided, the roots of both WT and *cipk18* had low and comparably basal amounts of ${\text{NH}}_{4}^{+}$ (Figure 3A). When excessive ${\text{NH}}_{4}^{+}$ was provided, a significant accumulation of ${\text{NH}}_{4}^{+}$ was observed in both lines (WT and *cipk18*). But the amount of ${\text{NH}}_{4}^{+}$ in the *cipk18* roots was significantly lower than that in the WT (Figure 3A). The enzymatic activities of GS and GOGAT for ${\text{NH}}_{4}^{+}$ assimilation in roots of both WT and *cipk18* were significantly increased due to external high ${\text{NH}}_{4}^{+}$. But there was no significant difference in the enzymatic activity between WT and *cipk18* (Figures 3B,C). These data indicated that the observed differential ammonium resistance between WT and *cipk18* lines could be a result of differences in root ${\text{NH}}_{4}^{+}$ accumulation, which was not associated with GS/GOGAT-dependent assimilation.

The difference in ${\text{NH}}_{4}^{+}$ accumulation in roots prompted us to investigate the changes in transcript abundance of OsAMT1s that encoded ammonium transporters. The expression of OsAMT1;1 and OsAMT1;3 was significantly suppressed to the same extent in both WT and *cipk18* by external high ${\text{NH}}_{4}^{+}$ (Figures 3D,F). The expression of OsAMT1;2 stayed unchanged in WT but was significantly downregulated in *cipk18* upon the external high ${\text{NH}}_{4}^{+}$ (Figure 3E). These findings suggest that the differences in ${\text{NH}}_{4}^{+}$ accumulation between WT and *cipk18* lines may be the result of direct or indirect involvement of OsCIPK18-mediated signaling in the transcriptional regulation of OsAMT1;2. Furthermore, this correlation prompted us to focus on the transcript changes in WT and *cipk18* during high ${\text{NH}}_{4}^{+}$ stress and identify the key genetic elements involved in differential ammonium resistance between the two lines.

### Genes Are Differentially Regulated Between WT and *cipk18*

**Figure 4:**
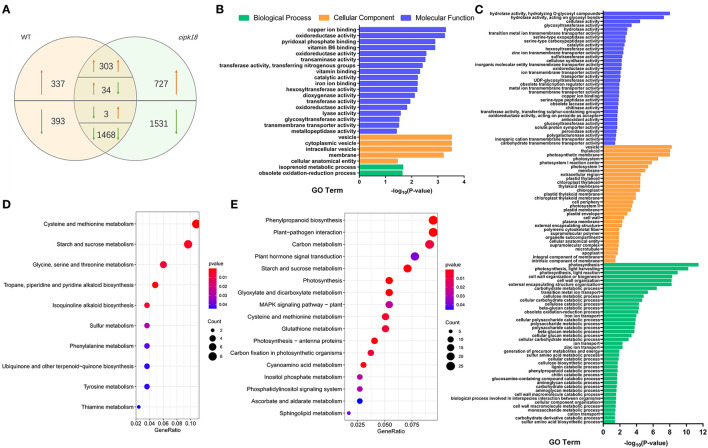
Transcriptomic analysis and enrich analysis of WT and *cipk18* in response to ammonium toxicity. **(A)** Venn diagram illustrates the number of DEGs in WT and *cipk18* under ammonium toxicity. **(B)** GO analysis of WT-specific DEGs. **(C)** GO analysis of *cipk18*-specific DEGs. **(D)** KEGG analysis of WT-specific DEGs. **(E)** KEGG analysis of *cipk18*-specific DEGs. DEGs, differentially expressed genes; GO, Gene Ontology; KEGG, Kyoto Encyclopedia of Genes and Genomes.

Transcriptome comparisons were performed between the control and ammonium stress treatments. In the WT line, a total of 2,538 excess ${\text{NH}}_{4}^{+}$-induced differentially expressed genes (DEGs) were identified, of which 674 DEGs were upregulated and 1,864 DEGs were downregulated in the presence of excess ${\text{NH}}_{4}^{+}$ (Figure 4A). In mutant *cipk18*, 4,066 excess ${\text{NH}}_{4}^{+}$-induced DEGs were identified, of which 43.6% (303 + 1,468 out of 4,066) had the same expression change trend in WT (Figure 4A). These overlapping DEGs most likely reflect regulatory networks induced by excess ${\text{NH}}_{4}^{+}$ independent of OsCIPK18. Notably, there were 730 (337 + 393) genes differentially expressed only in WT but not in *cipk18*, named WT-specific DEGs, reflecting ammonium toxicity-induced changes in gene expression dependent on OsCIPK18 (Figure 4A, Supplementary Table 1). These transcript changes may be one of the reasons for the phenotypic differences between WT and *cipk18*. In addition, 2,258 (727 + 1,531) DEGs were uniquely induced by high ${\text{NH}}_{4}^{+}$ in *cipk18* but not in WT, i.e., *cipk18*-specific DEGs, possibly reflecting an additional mechanism of ammonium resistance caused by knockdown of OsCIPK18 (Figure 4A, Supplementary Table 2). It is necessary to be concerned that there were 270 DEGs between WT and *cipk18*, even in the control conditions (Supplementary Figure 3A). These DEGs demonstrate important regulatory roles for OsCIPK18 in various signaling and physiological pathways in rice roots (Supplementary Figures 3C,D), which could explain the phenotypic differences observed in *cipk18* and WT lines (Figures 1A, 2A). There were 4.9% WT-specific DEGs (36 out of 730) and 2.1% *cipk18*-specific DEGs (48 out of 2258) overlapped with DEGs between WT and *cipk18* (Supplementary Figure 3B), and these overlapped DEGs may constitute factors that influence the response of *cipk18* to ammonium toxicity.

### Multiple Channels of ${\text{NH}}_{4}^{+}$ Transport Regulated by OsCIPK18

**Figure 5:**
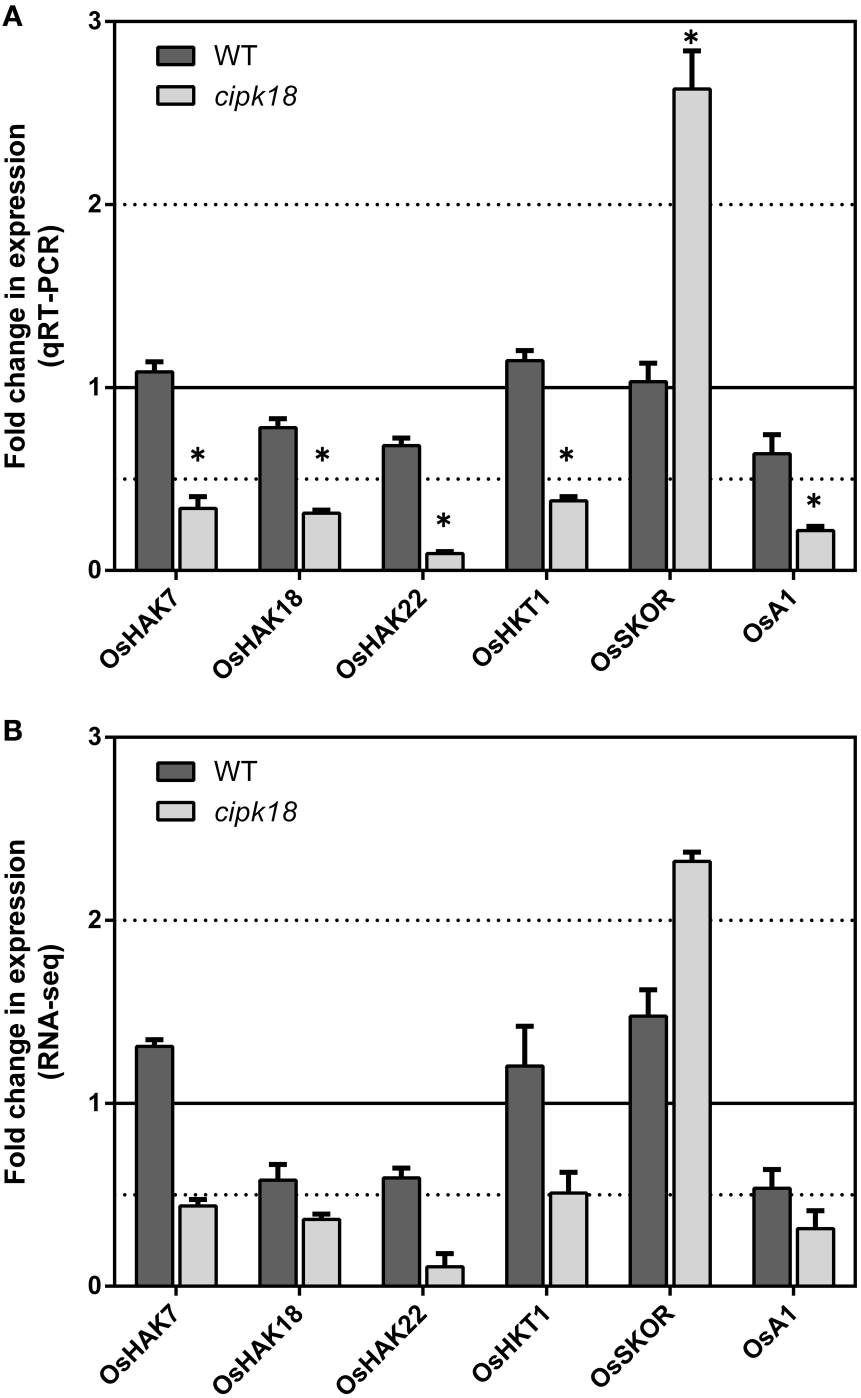
The expression of ${\text{NH}}_{4}^{+}$ transporters regulated by OsCIPK18 under ammonium toxicity. Fold changes in the expression of ${\text{NH}}_{4}^{+}$ transporters under ammonium toxicity, as determined by qRT-PCR **(A)** and RNA-seq analysis **(B)**. Data are means ± SDs. Significant differences using Student's *t*-test: *p < 0.05.

Ammonium could be transported through simple osmotic diffusion, non-selective cation channels, and potassium transport channels (Britto et al., 2014; Bittsánszky et al., 2015). Our previous expression analysis of AMT1 subfamily members had demonstrated that OsCIPK18 regulated ${\text{NH}}_{4}^{+}$ influx by altering the expression of OsAMT1;2 (Figure 3E). Using RNA-seq, we further identified four K^+^ transporters, OsHAK7 (*Os07g0669700*), OsHAK18 (*Os09g0563200*), OsHAK22 (*Os07g0102100*) (Banuelos et al., 2002), and OsHKT1 (*Os06g0701700*) (Yao et al., 2010), a plasma membrane H^+^-ATPase OsA1 (*Os03g0689300*), as well as an outward-rectifying shaker-like potassium channel OsSKOR (*Os04g0445000*) (Kim et al., 2015) in *cipk18*-specific DEGs (Figure 5). In *cipk18*, four K^+^ transporters were uniquely downregulated by excess ${\text{NH}}_{4}^{+}$ to reduce ${\text{NH}}_{4}^{+}$ uptake; meanwhile, OsSKOR was uniquely upregulated to increase ${\text{NH}}_{4}^{+}$ efflux (Figure 5). OsA1 could cooperatively improve N and C utilization and facilitates ammonium absorption in rice (Zhang M. et al., 2021). Its expression was significantly downregulated by excess ${\text{NH}}_{4}^{+}$ in *cipk18*, which may be one of the pathways through which OsCIPK18 regulates ${\text{NH}}_{4}^{+}$ uptake (Figure 5). In summary, we considered that the lower amount of ${\text{NH}}_{4}^{+}$ in *cipk18* could be the result of the simultaneous downregulation of OsAMT1;2, OsA1, OsHAK7/18/22, OsHKT1, and upregulation of OsSKOR. To further investigate how OsCIPK18 functions in the inhibition of root growth by ${\text{NH}}_{4}^{+}$, we investigated the functional distribution of WT-specific and *cipk18*-specific DEGs under ammonium toxicity, respectively, and tried to find the key genes.

### Ammonium Toxicity Response Process Dependent on OsCIPK18

WT-specific DEGs induced by excessive ${\text{NH}}_{4}^{+}$ differentially expressed only in WT represent an OsCIPK18-dependent ammonium toxicity response process. We explored the function of WT-specific DEGs based on GO and KEGG classification (Figures 4B,D). A total of 25 GO pathways and 10 KEGG pathways were enriched, mainly including transmembrane transporter activity, ion binding, vesicle, oxidoreductase activity, starch and sucrose metabolism, amino acid metabolism, and biosynthesis of small molecules, such as isoquinoline alkaloid and ubiquinone (Figures 4B,D).

**Figure 6:**
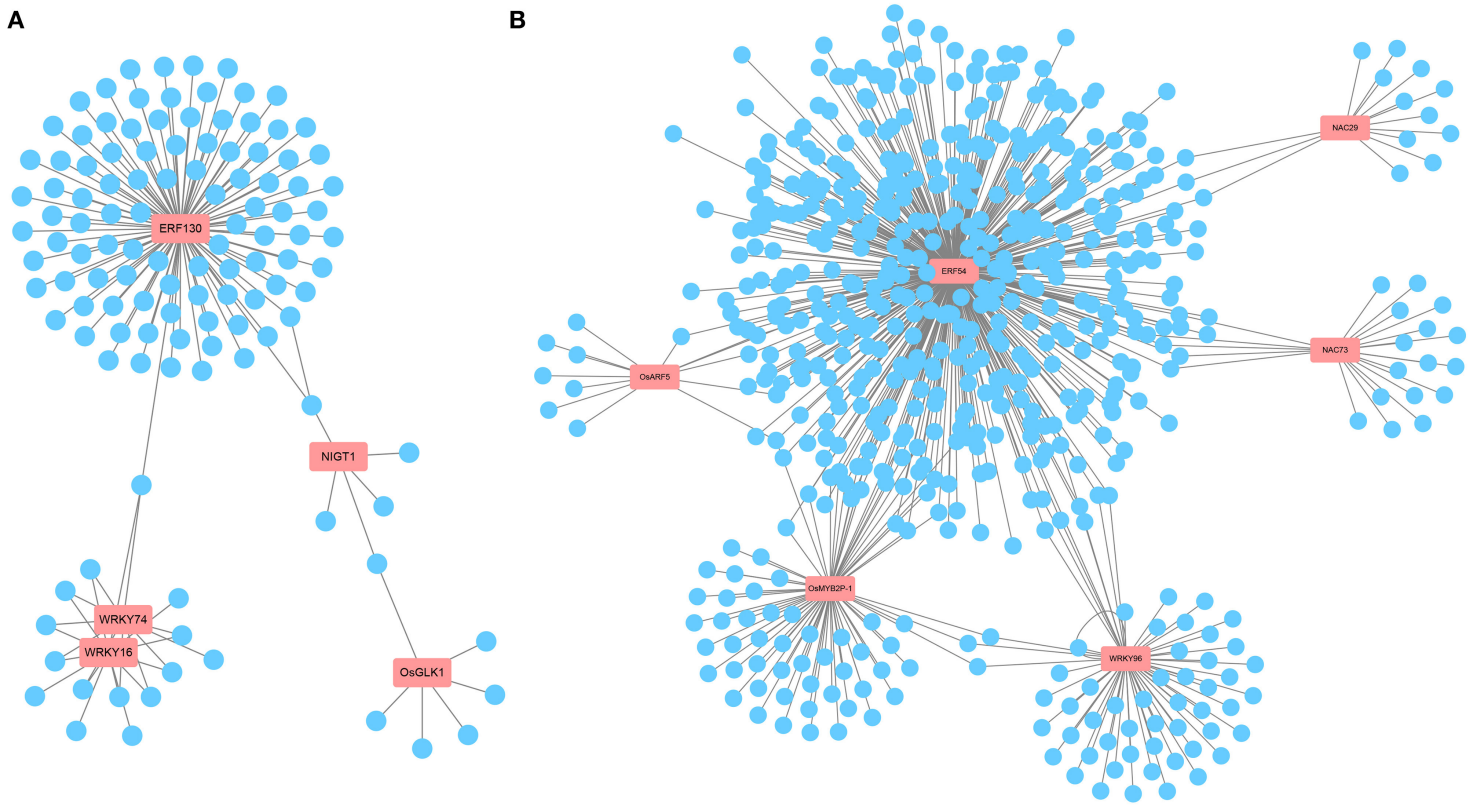
The key TFs and prediction network composed of DEGs. **(A)** The prediction network composed of WT-specific DEGs. **(B)** The prediction network composed of *cipk18*-specific DEGs. The genes in the red boxes are key TFs; blue circles represent predicted genes that may be activated or repressed in expression by TFs; gray lines represent possible transcriptional activation or repression. TFs, transcription factors.

Transcription factors, as key factors coordinating the expression of downstream genes involved in metabolic and developmental pathways, are important players in the response to ammonium toxicity (Kikuchi et al., 2000; Huang et al., 2015; Gu et al., 2017). Consistent with this, we found that multiple TF family members, such as AP2/ERF, MYB, WRKY, and NAC, were induced to be upregulated or downregulated upon exposure to excess ${\text{NH}}_{4}^{+}$ (Supplementary Table 1). In WT-specific DEGs, according to the predicted TF network using PlantRegMap, we found that five core TFs, OsERF130 (*Os05g0497200*), OsWRKY74 (*Os09g0334500*), OsWRKY16 (*Os01g0665500*), OsNIGT1 (*Os02g0325600*), and OsGLK1 (*Os06g0348800*), had possible interactions with multiple genes among the WT-specific DEGs (Figure 6A, Supplementary Figure 4, Supplementary Table 3). Among them, OsWRKY74 (Dai et al., 2016), whose expression was downregulated by ${\text{NH}}_{4}^{+}$ in WT, affected the elongation of roots and the increase of crown root number. This led us to speculate that ammonium toxicity signals might be transmitted through OsCIPK18 to OsWRKY74 to influence root development. Overall, this network revealed some key TFs that may be involved in root growth regulated by OsCIPK18 under ${\text{NH}}_{4}^{+}$ stress and regulatory relationships in WT-specific DEGs.

**Figure 7:**
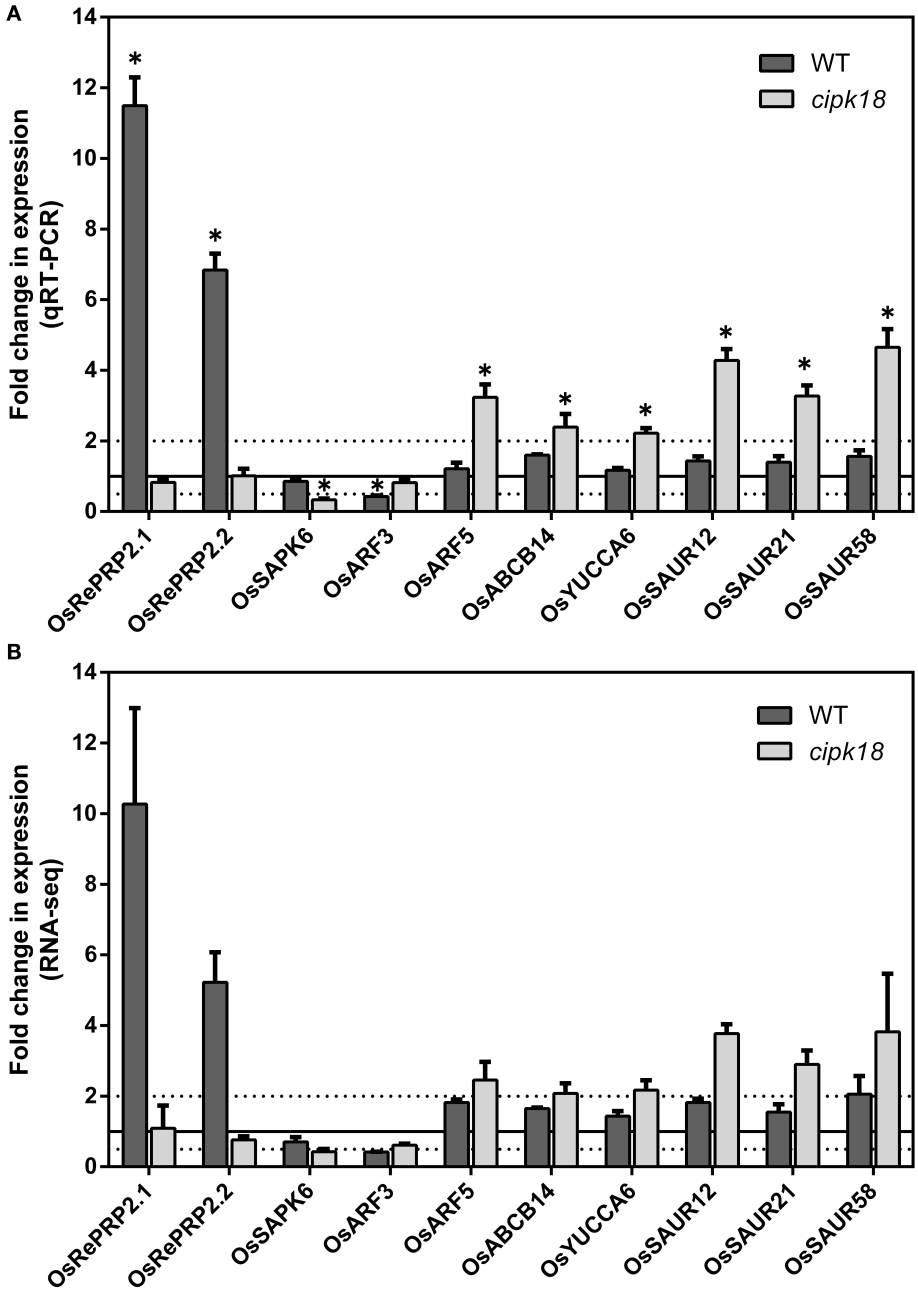
The expression of genes related to ABA and auxin signaling regulated by OsCIPK18 under ammonium toxicity. Fold changes in the expression of genes under ammonium toxicity, as determined by qRT-PCR **(A)** and RNA-seq analysis **(B)**. Data are means ± SDs. Significant differences using Student's *t*-test: *p < 0.05.

In addition, genes associated with ABA signaling in WT-specific DEGs attracted our attention. OsRePRP2.1 (*Os07g0418700*) and OsRePRP2.2 (*Os07g0418600*) were reported to be repressors of root cell expansion and were induced by ABA to be expressed in the elongation zone of roots (Tseng et al., 2013). In this study, their expression was significantly upregulated in WT (Figures 7A,B). Meanwhile, OsSAPK6 (*Os02g0551100*), encoded stress-activated protein kinase and involved in ABA signaling (Kobayashi et al., 2004), was uniquely downregulated in *cipk18* (Figures 7A,B). The expression changes of these genes implied that excess ${\text{NH}}_{4}^{+}$ activated ABA signaling and increased the expression of OsRePRP2.1/2.2 to inhibit root elongation. In contrast, due to the deletion of OsCIPK18, the expression of the ABA signaling factor OsSAPK6 was downregulated and the expression of OsRePRP2.1/2.2 was no longer upregulated, allowing the mutant *cipk18* to show greater resistance to ammonium.

### Mechanisms of Ammonium Resistance in the Mutant *cipk18*

In this study, *cipk18*-specific DEGs induced by excess ${\text{NH}}_{4}^{+}$ in *cipk18* were considered as components of the ammonium resistance mechanisms. Based on the GO and KEGG classification, the function of *cipk18*-specific DEGs is mainly related to ion transport, transmembrane transporter activity, cell wall macromolecule metabolic process, plant hormone signal transduction, cellular catabolic process, glutathione metabolism, sugar catabolic process, and photosystem (Figures 4C,E).

In *cipk18*-specific DEGs, we identified six key TFs that are likely to be the regulatory center of this part network, including OsERF54 (*Os01g0657400*), OsMYB2P-1 (*Os05g0140100*), OsWRKY96 (*Os12g0507300*), OsNAC29 (*Os08g0115800*), OsNAC73 (*Os01g0672100*), and ARF5 (*Os04g0664400*) (Figure 6B, Supplementary Figure 4, Supplementary Table 4). Among these genes, OsMYB2P-1, an R2R3 MYB TF engaged in phosphorus starvation response and regulation of root architecture in rice, was uniquely downregulated by ${\text{NH}}_{4}^{+}$ in *cipk18* (Dai et al., 2012). OsNAC29, a top-layer TF for secondary wall formation (Huang et al., 2015), was uniquely downregulated by ${\text{NH}}_{4}^{+}$ in *cipk18*, which was consistent with the cellulose synthesis-related pathway was enriched by GO classification. We, therefore, hypothesized that OsCIPK18 is associated with cell wall formation under ammonium toxicity.

Notably, OsARF5 is an auxin response factor whose expression is upregulated by excess ${\text{NH}}_{4}^{+}$ in the mutant *cipk18* (Figures 7A,B). This reminds us to be concerned about the role of auxin signaling in ammonium toxicity response. As we all know, ammonium feeding has been shown to suppress root auxin content (Kudoyarova et al., 1997; Britto and Kronzucker, 2002). In *cipk18*, we revealed that exposure to high ${\text{NH}}_{4}^{+}$ resulted in the upregulation of auxin synthesis and response gene expression, including the auxin influx transporter OsABCB14 (*Os04g0459000*) (Xu et al., 2014), the IAA synthetic pathway gene OsYUCCA6 (*Os07g0437000*) (Yamamoto et al., 2007), and three auxin-responsive SAUR gene family members [OsSAUR12 (*Os02g0769100*), OsSAUR21 (*Os04g0617050*), OsSAUR58 (*Os12g0626200*)] (Figures 7A,B) (Jain et al., 2006; Zhang T. et al., 2021). These results implied that knockdown of OsCIPK18 may have prevented the inhibition of auxin synthesis in roots by excess ${\text{NH}}_{4}^{+}$, allowing upregulation of auxin synthesis and response genes, thereby enhancing the resistance to ammonium toxicity in *cipk18*. Meanwhile, we found that OsARF3 (*Os01g0753500*) (Wang et al., 2007) was uniquely downregulated by excess ${\text{NH}}_{4}^{+}$ in WT-specific DEGs (Figures 7A,B), further supporting this hypothesis. Therefore, we hypothesized that seedling roots inhibit auxin signaling *via* OsCIPK18 in response to ammonium toxicity signals, resulting in inhibition of root growth in rice.

## Discussion

### The Mutant *cipk18* Shows ${\text{NH}}_{4}^{+}$-Resistance Phenotype

As a result of human intervention in the nitrogen cycle, including increasing soil nitrogen input and irrational fertilization practices in the biosphere, plants have to deal with unprecedented ${\text{NH}}_{4}^{+}$ stress (Gerendás et al., 1997; Britto and Kronzucker, 2002). Even though rice is a recognized ammonium-tolerant species and highly adapted to ${\text{NH}}_{4}^{+}$ as a nitrogen source, it is still threatened by ammonium toxicity (Balkos et al., 2010). Our study showed strong evidence that WT exhibited significant growth inhibition when it was grown under excessive ${\text{NH}}_{4}^{+}$ treatment, with few and short thick roots and reduced biomass (Figures 1, 2). However, a T-DNA insertion mutant *cipk18* exhibits enhanced resistance in response to ammonium toxicity as both biomass and root architecture showed that the inhibition of high ${\text{NH}}_{4}^{+}$ on roots growth in *cipk18* was less than that in WT (Figures 1, 2).

The CIPK family interacts with members of the CBL family in response to the ${\text{Ca}}_{2}^{+}$-mediated signaling pathway (Straub et al., 2017). OsCIPK18, as a member of the CIPK family, must also play an important role in rice growth and development, but this has not been reported yet. In this study, the mutant *cipk18* exhibited significant growth inhibition compared with WT (Figures 1, 2). Using GO and KEGG analysis, the 270 DEGs that were identified between WT and *cipk18* were associated with biological functions, such as signaling, response to stimuli, ion binding, and nitrogen metabolism (Supplementary Figures 3A,C,D). This discovery facilitates the study of the effects of OsCIPK18 in rice, but this study focuses on the role of OsCIPK18 in response to ammonium toxicity. The mutant *cipk18* shows ${\text{NH}}_{4}^{+}$-resistance phenotype, but mutants of two other members of the CIPK family, OsCIPK9 and OsCIPK23, were sensitive to ${\text{NH}}_{4}^{+}$ (Xuan et al., 2019), which suggested that the function of OsCIPK18 in response to ammonium toxicity might be opposite to that of OsCIPK9 and OsCIPK23.

### OsCIPK18 Affects ${\text{NH}}_{4}^{+}$ Transport

Traditional theories suggest that high intracellular ammonium concentration is one of the major causes of ammonium toxicity in higher plants, and there is also emerging evidence that acidic stress caused by excessive ${\text{NH}}_{4}^{+}$ assimilation is the primary cause (Hachiya et al., 2012, 2021; Esteban et al., 2016). In this study, intracellular ${\text{NH}}_{4}^{+}$ accumulation and GS/GOGAT-dependent ${\text{NH}}_{4}^{+}$ assimilation in roots were significantly increased by excess ${\text{NH}}_{4}^{+}$ in both WT and the mutant, but relatively low ${\text{NH}}_{4}^{+}$ accumulation was observed in *cipk18* (Figures 3A–C), suggesting that OsCIPK18 is involved in the ${\text{NH}}_{4}^{+}$ uptake without affecting ${\text{NH}}_{4}^{+}$ assimilation. In the presence of elevated external ${\text{NH}}_{4}^{+}$, ${\text{NH}}_{4}^{+}$ influx dependent on the high-affinity transport system is downregulated to prevent ammonium toxicity (Kronzucker et al., 2001; Beier et al., 2018; Kumar et al., 2020). For example, CBL1-CIPK23 phosphorylates and inactivates AMT1;1/1;2 in response to ammonium stress, thereby reducing ${\text{NH}}_{4}^{+}$ transport (Straub et al., 2017). In this study, expression of OsAMT1;1 and OsAMT1;3 was significantly downregulated by excess ${\text{NH}}_{4}^{+}$ in both WT and mutant (Figures 3D,E). However, in *cipk18*, the expression of OsAMT1;2, OsHAK7, OsHAK18, OsHAK22, OsHKT1, and OsA1, which are reported to transport ${\text{NH}}_{4}^{+}$, were all inhibited by excess ${\text{NH}}_{4}^{+}$ (Figures 3E, 5A,B). Changes in the expression of these genes coincided with reduced ammonium accumulation in the mutant, suggesting that OsCIPK18 plays an important role in maintaining the expression of ammonium transporters at the transcriptional level during the response to ammonium toxicity.

### Ammonium Toxicity-Induced Transcriptional Regulatory Network Dependent on OsCIPK18

Apart from the regulation of ${\text{NH}}_{4}^{+}$ uptake, it is not known how OsCIPK18 regulates root growth under ammonium stress. Transcriptome-wide analysis facilitated our observation of high ${\text{NH}}_{4}^{+}$-induced changes in gene expression associated with root growth at the genome-wide level. Sun et al. used RNA-seq to reveal the spatiotemporal specificity of gene expression in rice after high ammonium treatment for 4 and 12 h, highlighting the role of TFs and phytohormones in ammonium resistance (Sun et al., 2017). In our data, WT-specific DEGs and *cipk18*-specific DEGs were identified as differential genes involved in response to ammonium toxicity and located downstream of OsCIPK18 (Figure 4A). Strategies for coping with salt stress in plants include adjusting ATP formation and enhancing energy metabolism (Zhao et al., 2013). Genes related to amino acid metabolism, starch, and sucrose metabolism, as well as oxidoreductase, lysozyme, and many other enzymatic activities are regulated by OsCIPK18 under ammonium toxicity (Figures 4B–E). Recent evidence suggests that the biosynthesis of vitamin B6 significantly improves root tolerance to ammonium (Liu et al., 2022). Our data show that genes associated with vitamin B6 binding are also regulated by OsCIPK18 (Figure 4B). The above evidence highlights the fundamental function of OsCIPK18 in the ammonium toxicity response.

**Figure 8:**
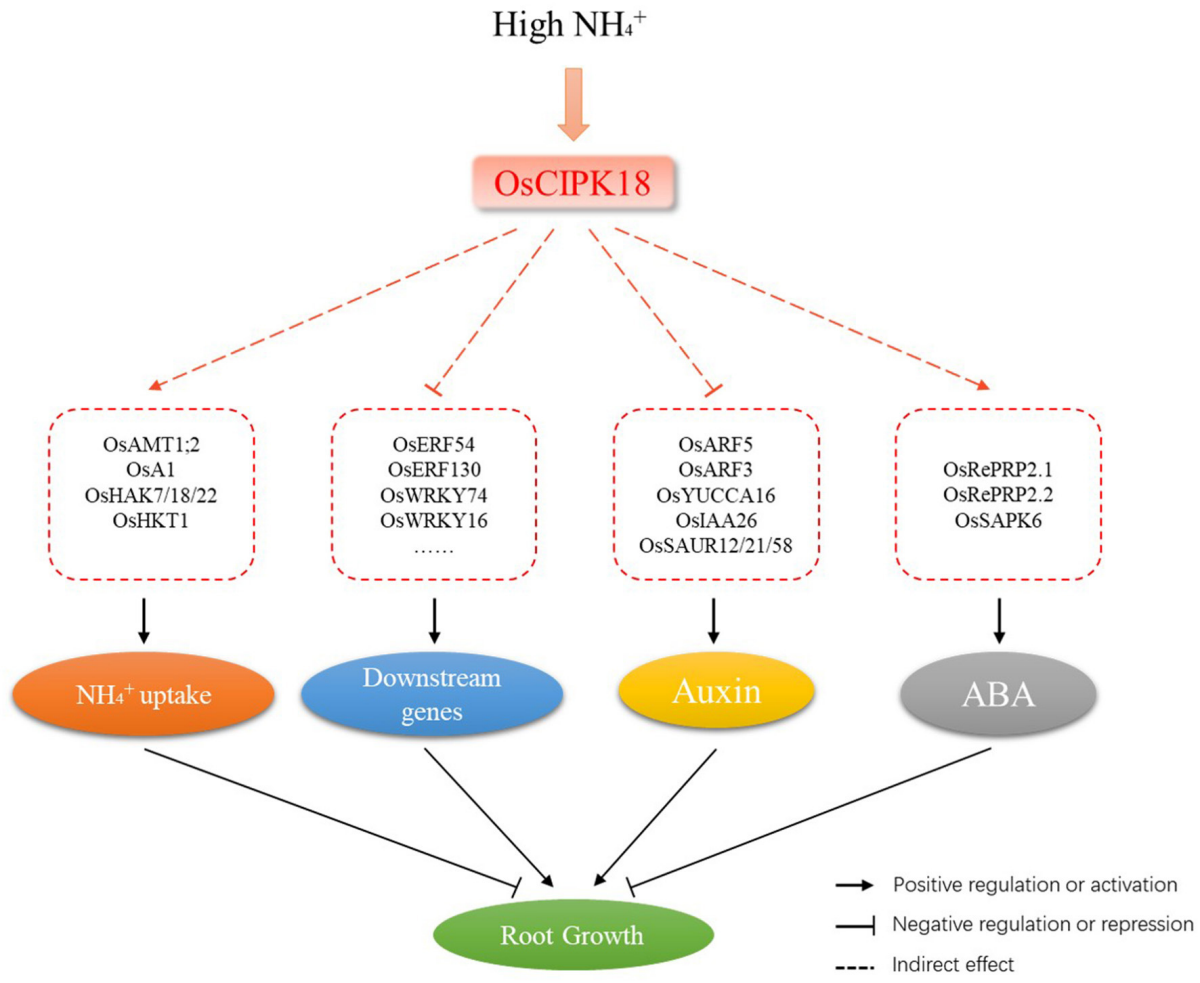
A schematic model of the involvement of OsCIPK18 in ammonium toxicity in rice seedling roots. osCIPK18 directly or indirectly regulates the expression of several genes that affect ${\text{NH}}_{4}^{+}$ accumulation, the ABA and auxin signaling pathways, and the transcription factor regulatory network, ultimately regulating root growth.

Several core TFs were identified in WT-specific and *cipk18*-specific DEGs that could be directly or indirectly regulated by OsCIPK18 under ammonium stress (Figures 6A,B). Members of the AP2/ERF family are associated with plant defense programs against abiotic stresses and share a conserved DNA-binding domain that activates downstream gene expression by binding specifically to *cis*-acting elements in the promoters of abiotic stress-responsive genes (Mizoi et al., 2012). In this study, two ERF family genes (OsERF54, OsERF130) were found to be at the center of the transcriptional regulatory network involved in ammonium toxicity by OsCIPK18 (Figures 6A,B), suggesting that OsCIPK18 might regulate the expression of ERF genes to further regulate downstream genes in response to ammonium toxicity (Figure 8). WRKY TFs in rice are rapidly induced in response to abiotic stresses, such as salinity, aluminum, drought, and osmotic stress, to regulate developmental processes, such as seed development, root growth, and leaf senescence (Ross et al., 2007). Three key genes (OsWRKY16, OsWRKY74, and OsWRKY96) belonging to the WRKY family were identified in WT-specific DEGs or *cipk18*-specific DEGs (Figures 6A,B). Among them, OsWRKY74 was reported to be involved in the response of Pi starvation, Fe starvation, as well as cold stress, and promote the elongation of primary and adventitious roots (Dai et al., 2016). In this study, inhibition of OsWRKY74 by excess ${\text{NH}}_{4}^{+}$ was lost in the mutant, suggesting that OsWRKY74 lies downstream of OsCIPK18 in regulating root growth under ammonium toxicity. In addition, members of the NAC, MYB, and other families were identified to be regulated by OsCIPK18 and involved in the inhibition of root growth in response to ammonium toxicity. These TFs and their interacting functional genes together form a response network of excess ${\text{NH}}_{4}^{+}$ regulated by OsCIPK18, which will contribute to the understanding of the process of ammonium toxicity production.

### Role of OsCIPK18 in the Regulation of Auxin and ABA Signaling by Ammonium Toxicity

Phytohormones play an integral role in the regulatory network of response to ammonium toxicity in plants (Zou et al., 2013; Lei et al., 2016; Di et al., 2018; Sun et al., 2020). Excessive ammonium supply reduced free IAA content in the roots and significantly accelerated tissue ABA accumulation (Di et al., 2018; Sun et al., 2020). In the mutant *cipk18*, an auxin biosynthetic gene OsYUCCA6, an auxin influx transporter OsABCB14, and auxin-related response genes (OsARF5, OsSAUR12, OsSAUR21, and OsSAUR58) were specifically upregulated by excess ${\text{NH}}_{4}^{+}$ (Figures 7A,B). This demonstrated that both auxin synthesis and response pathway were activated in *cipk18*, suggesting that excessive ammonium suppressed root growth through direct or indirect regulation of the auxin synthesis gene by OsCIPK18, reducing IAA content and inhibiting the auxin response pathway (Figure 8). In the ABA signaling pathway, OsRePRP2.1 and OsRePRP2.2 were able to be activated by ABA and inhibited cell expansion (Tseng et al., 2013), and OsSAPK6 expression was significantly increased by ABA treatment (Chae et al., 2007). Under ammonium stress, OsSAPK6 was uniquely downregulated, and OsRePRP2.1/2.2 were no longer upregulated in *cipk18*, implying that knockdown of OsCIPK18 results in blocked ABA signaling, thereby reducing the inhibitory effect of ammonium toxicity on root growth (Figures 7A,B). These data reveal that OsCIPK18 is a key node in hormone signaling under ammonium stress, which provides great insight into the involvement of key phytohormones in rice in response to ${\text{NH}}_{4}^{+}$ stress.

## Data Availability Statement

The datasets presented in this study can be found in online repositories. The names of the repository/repositories and accession number(s) can be found at: BioProject ID:PRJNA776549, https://www.ncbi.nlm.nih.gov/bioproject/PRJNA776549.

## Author Contributions

JinY designed the experiments. YL and XP directed the experiments. JinY and TS performed most of the experiments and analyses. YQ, GZ, and JiaY helped with the quantification of phenotypes. TS wrote the manuscript. JinY, TW, and HZ revised the manuscript. All authors discussed the results and contributed to the finalization of the manuscript. All authors contributed to the article and approved the submitted version.

## Funding

This study was supported by the National Natural Science Foundation of China (Nos. 31760377, 31960124, and 32172074), the National Special Key Project for Transgenic Breeding (Grant No. 2016ZX08001001), and the National Key Research and Development Program of China (2016YFD0100400).

## Conflict of Interest

The authors declare that the research was conducted in the absence of any commercial or financial relationships that could be construed as a potential conflict of interest.

## Publisher's Note

All claims expressed in this article are solely those of the authors and do not necessarily represent those of their affiliated organizations, or those of the publisher, the editors and the reviewers. Any product that may be evaluated in this article, or claim that may be made by its manufacturer, is not guaranteed or endorsed by the publisher.

## Supplementary Material

Supplementary Figure 1 Identification of the T-DNA insertion site of *cipk18*. **(A)** A schematic map of the T-DNA insertion sites on the genomic regions of OsCIPK18. **(B)** Detection of the flanking sequence of the T-DNA insertion site in the mutant *via* PCR/RE assay.

Supplementary Figure 2 The mutant *cipk18* shows resistance to excess ${\text{NH}}_{4}^{+}$. **(A–D)** Rice seedlings phenotypes **(A,B)**, root FW **(C)**, and radicle length **(D)** of 5-day-old WT, *cipk18*, and three complementary materials of *cipk18* (com1, com2, and com3) under treatments with ddH_2_O and 4 mM NH_4_Cl, respectively. Scale bar = 1 cm. **(E)** Compared with the control, relative root growth of the five lines under 4 mM NH_4_Cl. Data are means ± SDs, significant differences using Student's *t*-test: ^*^*p* < 0.05.

Supplementary Figure 3 Differentially expressed genes between WT and *cipk18* cultured in ddH_2_O. **(A)** Volcano plot of DEGs between WT and *cipk18*. **(B)** Venn diagram of DEGs between WT and *cipk18*, WT-specific DEGs, and *cipk18*-specific DEGs. **(C)** GO analysis of DEGs between WT and *cipk18*. **(D)** KEGG analysis of DEGs between WT and *cipk18*.

Supplementary Figure 4 The expression of key TF genes regulated by OsCIPK18 under ammonium toxicity. Fold changes in the expression of genes under ammonium toxicity, as determined by qRT-PCR **(A)** and RNA-seq analysis **(B)**. Data are means ± SDs. Significant differences using Student's *t*-test: ^*^*p* < 0.05.

Supplementary Table 1 Expression of WT-specific differentially expressed genes.

Supplementary Table 2 Expression of *cipk18*-specific differentially expressed genes.

Supplementary Table 3 Predicted transcriptional regulatory networks in WT-specific DEGs under ammonium toxicity.

Supplementary Table 4 Predicted transcriptional regulatory networks in *cipk18*-specific DEGs under ammonium toxicity.

Supplementary Table 5 Primers used in this study.

## References

[B1] Alencar V. Lobo A. K. M. Carvalho F. E. L. Silveira J. A. G. (2019). High ammonium supply impairs photosynthetic efficiency in rice exposed to excess light. Photosyn. Res. 140, 321–335. 10.1007/s11120-019-00614-z 30694432

[B2] Balkos K. D. Britto D. T. Kronzucker H. J. (2010). Optimization of ammonium acquisition and metabolism by potassium in rice (Oryza sativa L. cv. IR-72). Plant Cell Environ. 33, 23–34. 10.1111/j.1365-3040.2009.02046.x 19781010

[B3] Banuelos M. A. Garciadeblas B. Cubero B. Rodríguez-Navarro A. (2002). Inventory and functional characterization of the HAK potassium transporters of rice. Plant Physiol. 130, 784–795. 10.1104/pp.007781 12376644 PMC166606

[B4] Beier M. P. Obara M. Taniai A. Sawa Y. Ishizawa J. Yoshida H.. (2018). Lack of ACTPK1, an STY kinase, enhances ammonium uptake and use, and promotes growth of rice seedlings under sufficient external ammonium. Plant J. 93, 992–1006. 10.1111/tpj.13824 29356222

[B5] Bittsánszky A. Pilinszky K. Gyulai G. Komives T. (2015). Overcoming ammonium toxicity. Plant Sci. 231, 184–190. 10.1016/j.plantsci.2014.12.005 25576003

[B6] Bolger A. M. Lohse M. Usadel B. (2014). Trimmomatic: a flexible trimmer for Illumina sequence data. Bioinformatics 30, 2114–2120. 10.1093/bioinformatics/btu170 24695404 PMC4103590

[B7] Britto D. T. Balkos K. D. Becker A. Coskun D. Huynh W. Q. Kronzucker H. J. (2014). Potassium and nitrogen poising: physiological changes and biomass gains in rice and barley. Canad. J. Plant Sci. 94, 1085–1089. 10.4141/cjps2013-143

[B8] Britto D. T. Kronzucker H. J. (2002). toxicity in higher plants: a critical review. J. Plant Physiol. 159, 567–584. 10.1078/0176-1617-0774

[B9] Chae M.-J. Lee J.-S. Nam M.-H. Cho K. Hong J.-Y. Yi S.-A.. (2007). A rice dehydration-inducible SNF1-related protein kinase 2 phosphorylates an abscisic acid responsive element-binding factor and associates with ABA signaling. Plant Mol. Biol. 63, 151–169. 10.1007/s11103-006-9079-x 16977424

[B10] Chen C. Chen H. Zhang Y. Thomas H. R. Frank M. H. He Y.. (2020). TBtools: an integrative toolkit developed for interactive analyses of big biological data. Mol. Plant 13, 1194–1202. 10.1016/j.molp.2020.06.009 32585190

[B11] Chen G. Guo S. Kronzucker H. J. Shi W. (2013). Nitrogen use efficiency (NUE) in rice links to toxicity and futile cycling in roots. Plant Soil 369, 351–363. 10.1007/s11104-012-1575-y

[B12] Cheng X. Sardana R. K. Altosaar I. (1998). Rice transformation by agrobacterium infection, in Recombinant Proteins from Plants, Vol. 3, eds Cunningham C. Porter A. J. R. (Totowa: Humana Press) 1–9. 10.1007/978-1-60327-260-5_1

[B13] Dai X. Wang Y. Yang A. Zhang W. H. (2012). OsMYB2P-1, an R2R3 MYB transcription factor, is involved in the regulation of phosphate-starvation responses and root architecture in rice. Plant Physiol. 159, 169–183. 10.1104/pp.112.194217 22395576 PMC3375959

[B14] Dai X. Wang Y. Zhang W. H. (2016). OsWRKY74, a WRKY transcription factor, modulates tolerance to phosphate starvation in rice. J. Exp. Bot. 67, 947–960. 10.1093/jxb/erv515 26663563 PMC4737085

[B15] Dave G. Nilsson E. (2005). Increased reproductive toxicity of landfill leachate after degradation was caused by nitrite. Aquatic Toxicol. 73, 11–30. 10.1016/j.aquatox.2005.02.006 15892989

[B16] Di D. Sun L. Zhang X. Li G. Kronzucker H. J. Shi W. (2018). Involvement of auxin in the regulation of ammonium tolerance in rice (Oryza sativa L.). Plant Soil 432, 373–387. 10.1007/s11104-018-3813-4

[B17] Dobin A. Davis C. A. Schlesinger F. Drenkow J. Zaleski C. Jha S.. (2013). STAR: ultrafast universal RNA-seq aligner. Bioinformatics 29, 15–21. 10.1093/bioinformatics/bts635 23104886 PMC3530905

[B18] Esteban R. Ariz I. Cruz C. Moran J. F. (2016). Review: mechanisms of ammonium toxicity and the quest for tolerance. Plant Sci. 248, 92–101. 10.1016/j.plantsci.2016.04.008 27181951

[B19] Forde B. G. (2002). Local and long-range signaling pathways regulating plant responses to nitrate. Annu. Rev. Plant Biol. 53, 203–224. 10.1146/annurev.arplant.53.100301.135256 12221973

[B20] Gerendás J. Zhu Z. Bendixen R. Ratcliffe R. G. Sattelmacher B. (1997). Physiological and biochemical processes related to ammonium toxicity in higher plants. J. Plant Nutr. Soil Sci. 160, 239–251. 10.1002/jpln.19971600218

[B21] Gu M. Zhang J. Li H. Meng D. Li R. Dai X.. (2017). Maintenance of phosphate homeostasis and root development are coordinately regulated by MYB1, an R2R3-type MYB transcription factor in rice. J. Exp. Bot. 68, 3603–3615. 10.1093/jxb/erx174 28549191 PMC5853628

[B22] Hachiya T. Inaba J. Wakazaki M. Sato M. Toyooka K. Miyagi A.. (2021). Excessive ammonium assimilation by plastidic glutamine synthetase causes ammonium toxicity in *Arabidopsis thaliana*. Nat. Commun. 12, 4944. 10.1038/s41467-021-25238-7 34400629 PMC8367978

[B23] Hachiya T. Watanabe C. K. Fujimoto M. Ishikawa T. Takahara K. Kawai-Yamada M.. (2012). Nitrate addition alleviates ammonium toxicity without lessening ammonium accumulation, organic acid depletion and inorganic cation depletion in *Arabidopsis thaliana* shoots. Plant Cell Physiol. 53, 577–591. 10.1093/pcp/pcs012 22318863

[B24] Hashimoto K. Kudla J. (2011). Calcium decoding mechanisms in plants. Biochimie 93, 2054–2059. 10.1016/j.biochi.2011.05.019 21658427

[B25] Hirano T. Satoh Y. Ohki A. Takada R. Arai T. Michiyama H. (2008). Inhibition of ammonium assimilation restores elongation of seminal rice roots repressed by high levels of exogenous ammonium. Physiol. Plant. 134, 183–190. 10.1111/j.1399-3054.2008.01117.x 18419739

[B26] Hu H. C. Wang Y. Y. Tsay Y. F. (2009). AtCIPK8, a CBL-interacting protein kinase, regulates the low-affinity phase of the primary nitrate response. Plant J. 57, 264–278. 10.1111/j.1365-313X.2008.03685.x 18798873

[B27] Huang D. Wang S. Zhang B. Shang-Guan K. Shi Y. Zhang D.. (2015). A gibberellin-mediated DELLA-NAC signaling cascade regulates cellulose synthesis in rice. Plant Cell 27, 1681–1696. 10.1105/tpc.15.00015 26002868 PMC4498200

[B28] Jain M. Tyagi A. K. Khurana J. P. (2006). Genome-wide analysis, evolutionary expansion, and expression of early auxin-responsive SAUR gene family in rice (Oryza sativa). Genomics 88, 360–371. 10.1016/j.ygeno.2006.04.008 16707243

[B29] Jeon J. Lee S. Jung K. Jun S. Jeong D. Lee J.. (2000). T-DNA insertional mutagenesis for functional genomics in rice. Plant J. 22, 561–570. 10.1046/j.1365-313x.2000.00767.x 10886776

[B30] Kikuchi K. Ueguchi-Tanaka M. Yoshida K. T. Nagato Y. Hirano H. Y. (2000). Molecular analysis of the NAC gene family in rice. Mol. General Genet. 262, 1047–1051. 10.1007/PL00008647 10660065

[B31] Kim H. Y. Choi E.-H. Min M. K. Hwang H. Moon S.-J. Yoon I.. (2015). Differential gene expression of two outward-rectifying shaker-like potassium channels OsSKOR and OsGORK in rice. J. Plant Biol. 58, 230–235. 10.1007/s12374-015-0070-4

[B32] Kobayashi Y. Yamamoto S. Minami H. Hattori K. T. (2004). Differential activation of the rice sucrose Nonfermenting1-related protein Kinase2 family by hyperosmotic stress and abscisic acid. Plant Cell 16, 1163–1177. 10.1105/tpc.019943 15084714 PMC423207

[B33] Konishi N. Ma J. F. (2021). Three polarly localized ammonium transporter 1 members are cooperatively responsible for ammonium uptake in rice under low ammonium condition. New Phytol. 232, 1778–1792. 10.1111/nph.17679 34392543

[B34] Kronzucker H. J. Britto D. T. Davenport R. J. Tester M. (2001). Ammonium toxicity and the real cost of transport. Trends Plant Sci. 6, 335–337. 10.1016/S1360-1385(01)02022-2 11495764

[B35] Krouk G. Lacombe B. Bielach A. Perrine-Walker F. Malinska K. Mounier E.. (2010). Nitrate-regulated auxin transport by NRT1.1 defines a mechanism for nutrient sensing in plants. Dev. Cell 18, 927–937. 10.1016/j.devcel.2010.05.008 20627075

[B36] Kudoyarova G. R. Farkhutdinov R. G. Veselov S. Y. (1997). Comparison of the effects of nitrate and ammonium forms of nitrogen on auxin content in roots and the growth of plants under different temperature conditions. Plant Growth Regul. 23, 207–208. 10.1023/A:1005990725068

[B37] Kumar V. Kim S. H. Priatama R. A. Jeong J. H. Adnan M. R. Saputra B. A.. (2020). suppresses -dependent lateral root growth and alters gene expression and gravity response in OsAMT1 RNAi mutants of rice (Oryza sativa). J. Plant Biol. 63, 391–407. 10.1007/s12374-020-09263-5

[B38] Lei D. Li Y. Ying W. Gao L. Wang M. Francois C.. (2016). Root ABA accumulation enhances rice seedling drought tolerance under ammonium supply: interaction with aquaporins. Front. Plant Sci. 7, 1206. 10.3389/fpls.2016.01206 27559341 PMC4979525

[B39] Li B. Dewey C. N. (2011). RSEM: accurate transcript quantification from RNA-Seq data with or without a reference genome. BMC Bioinformatics 12, 323. 10.1186/1471-2105-12-323 21816040 PMC3163565

[B40] Li B. Li G. Kronzucker H. J. Baluska F. Shi W. (2014). Ammonium stress in Arabidopsis: signaling, genetic loci, and physiological targets. Trends Plant Sci. 19, 107–114. 10.1016/j.tplants.2013.09.004 24126103

[B41] Li B. Li Q. Xiong L. Kronzucker H. J. Kramer U. Shi W. (2012). Arabidopsis plastid AMOS1/EGY1 integrates abscisic acid signaling to regulate global gene expression response to ammonium stress. Plant Physiol. 160, 2040–2051. 10.1104/pp.112.206508 23064408 PMC3510130

[B42] Liu Y. Maniero R. A. Giehl R. F. H. Melzer M. Steensma P. Krouk G.. (2022). PDX1.1-dependent biosynthesis of vitamin B6 protects roots from ammonium-induced oxidative stress. Mol. Plant. 15, 1–20. 10.1016/j.molp.2022.01.012 35063660

[B43] Liu Y. Wirén N. V. (2017). Ammonium as a signal for physiological and morphological responses in plants. J. Exp. Bot. 68, 2581–2592. 10.1093/jxb/erx086 28369490

[B44] Lobet G. Draye L. P. (2011). A novel image-analysis toolbox enabling quantitative analysis of root system architecture. Plant Physiol. 157, 29–39. 10.1104/pp.111.179895 21771915 PMC3165877

[B45] Meier M. Liu Y. Lay-Pruitt K. S. Takahashi H. von Wirén N. (2020). Auxin-mediated root branching is determined by the form of available nitrogen. Nat. Plants 6, 1136–1145. 10.1038/s41477-020-00756-2 32917974

[B46] Miflin B. J. Habash D. Z. (2002). The role of glutamine synthetase and glutamate dehydrogenase in nitrogen assimilation and possibilities for improvement in the nitrogen utilization of crops. J. Exp. Bot. 53, 979–987. 10.1093/jexbot/53.370.979 11912240

[B47] Miller A. J. Cramer M. D. (2005). Root nitrogen acquisition and assimilation. Plant Soil 274, 1–36. 10.1007/1-4020-4099-7_1

[B48] Mizoi J. Shinozaki K. Yamaguchi-Shinozaki K. (2012). AP2/ERF family transcription factors in plant abiotic stress responses. Biochim. Biophys. Acta 1819, 86–96. 10.1016/j.bbagrm.2011.08.004 21867785

[B49] Romano N. Zeng C. (2007). Acute toxicity of ammonia and its effects on the haemolymph osmolality, ammonia-N, pH and ionic composition of early juvenile mud crabs, Scylla serrata (Forskl). Comp. Biochem. Physiol. A Mol. Integr. Physiol. 148, 278–285. 10.1016/j.cbpa.2007.04.018 17540593

[B50] Ross C. A. Liu Y. Shen Q. J. (2007). The WRKY gene family in rice (Oryza sativa). J. Integr. Plant Biol. 49, 827–842. 10.1111/j.1744-7909.2007.00504.x30175125

[B51] Sánchez-Barrena M. J. Martínez-Ripoll M. Zhu J.-K. Albert A. (2005). The structure of the *Arabidopsis thaliana* SOS3: molecular mechanism of sensing calcium for salt stress response. J. Mol. Biol. 345, 1253–1264. 10.1016/j.jmb.2004.11.025 15644219

[B52] Straub T. Ludewig U. Neuhauser B. (2017). The kinase CIPK23 inhibits ammonium transport in *Arabidopsis thaliana*. Plant Cell 29, 409–422. 10.1105/tpc.16.00806 28188265 PMC5354196

[B53] Sun L. Di D. Li G. Kronzucker H. J. Shi W. (2017). Spatio-temporal dynamics in global rice gene expression (Oryza sativa L.) in response to high ammonium stress. J. Plant Physiol. 212, 94–104. 10.1016/j.jplph.2017.02.006 28282528

[B54] Sun L. Di D. W. Li G. Kronzucker H. J. Wu X. Shi W. (2020). Endogenous ABA alleviates rice ammonium toxicity by reducing ROS and free ammonium via regulation of the SAPK9-bZIP20 pathway. J. Exp. Bot. 71, 4562–4577. 10.1093/jxb/eraa076 32064504 PMC7475098

[B55] Tian T. Liu Y. Yan H. You Q. Yi X. Du Z.. (2017). agriGO v2.0: a GO analysis toolkit for the agricultural community, 2017 update. Nucleic Acids Res. 45, W122–W129. 10.1093/nar/gkx382 28472432 PMC5793732

[B56] Tseng I. C. Hong C. Y. Yu S. M. Ho T. (2013). Abscisic acid- and stress-induced highly proline-rich glycoproteins regulate root growth in rice. Plant Physiol. 163, 118–134. 10.1104/pp.113.217547 23886623 PMC3762635

[B57] Wang D. Pei K. Fu Y. Sun Z. Li S. Liu H.. (2007). Genome-wide analysis of the auxin response factors (ARF) gene family in rice (*Oryza sativa*). Gene 394, 13–24. 10.1016/j.gene.2007.01.006 17408882

[B58] Xu J. Li H.-D. Chen L.-Q. Wang Y. Liu L.-L. He L.. (2006). A protein kinase, interacting with two calcineurin B-like proteins, regulates K^+^ transporter AKT1 in arabidopsis. Cell 125, 1347–1360. 10.1016/j.cell.2006.06.011 16814720

[B59] Xu Y. Zhang S. Guo H. Wang S. Xu L. Li C.. (2014). OsABCB14 functions in auxin transport and iron homeostasis in rice (*Oryza sativa* L.). Plant J. 79, 106–117. 10.1111/tpj.12544 24798203

[B60] Xuan Y. H. Kumar V. Han X. Kim S. H. Jeong J. H. Kim C. M.. (2019). CBL-INTERACTING PROTEIN KINASE 9 regulates ammonium-dependent root growth downstream of IDD10 in rice (*Oryza sativa*). Ann. Bot. 124, 947–960. 10.1093/aob/mcy242 30715138 PMC6881222

[B61] Yamamoto Y. Kamiya N. Morinaka Y. Matsuoka M. Sazuka T. (2007). Auxin biosynthesis by the YUCCA genes in rice. Plant Physiol. 143, 1362–1371. 10.1104/pp.106.091561 17220367 PMC1820910

[B62] Yao X. Horie T. Xue S. Leung H. Y. Katsuhara M. Brodsky D. E.. (2010). Differential sodium and potassium transport selectivities of the rice OsHKT2;1 and OsHKT2;2 transporters in plant cells. Plant Physiol. 152, 341–355. 10.1104/pp.109.145722 19889878 PMC2799368

[B63] Yong H. C. Pandey G. K. Grant J. J. Batistic O. Li L. Kim B. G.. (2010). Two calcineurin B-like calcium sensors, interacting with protein kinase CIPK23, regulate leaf transpiration and root potassium uptake in Arabidopsis. Plant J. 52, 223–239. 10.1111/j.1365-313X.2007.03236.x 17922773

[B64] Yu G. Wang L. G. Han Y. He Q. Y. (2012). clusterProfiler: an R package for comparing biological themes among gene clusters. OMICS 16, 284–287. 10.1089/omi.2011.0118 22455463 PMC3339379

[B65] Yutaka S. Akira I. Satomi S. von W. N. Tomoyuki Y. Junji Y. (2003). Distinct expression and function of three ammonium transporter genes (OsAMT1;1 – 1;3) in rice. Plant Cell Physiol. 7, 726–734. 10.1093/pcp/pcg083 12881500

[B66] Zhang M. Wang Y. Chen X. Xu F. Ding M. Ye W.. (2021). Plasma membrane H^+^-ATPase overexpression increases rice yield via simultaneous enhancement of nutrient uptake and photosynthesis. Nat. Commun. 12, 735. 10.1038/s41467-021-20964-4 33531490 PMC7854686

[B67] Zhang T. You J. Zhang Y. Yao W. Chen W. Duan Q.. (2021). LF1 regulates the lateral organs polarity development in rice. New Phytol. 231, 1265–1277. 10.1111/nph.17220 33469925

[B68] Zhao Q. Zhang H. Wang T. Chen S. Dai S. (2013). Proteomics-based investigation of salt-responsive mechanisms in plant roots. J. Proteomics 82, 230–253. 10.1016/j.jprot.2013.01.024 23385356

[B69] Zhu Y. Di T. Xu G. Chen X. Zeng H. Yan F.. (2009). Adaptation of plasma membrane H(^+^)-ATPase of rice roots to low pH as related to ammonium nutrition. Plant Cell Environ. 32, 1428–1440. 10.1111/j.1365-3040.2009.02009.x 19558410

[B70] Zou N. Li B. Chen H. Su Y. Kronzucker H. J. Xiong L.. (2013). GSA-1/ARG1 protects root gravitropism in Arabidopsis under ammonium stress. New Phytol. 200, 97–111. 10.1111/nph.12365 23782229

[B71] Zou N. Li B. Dong G. Kronzucker H. J. Shi W. (2012). Ammonium-induced loss of root gravitropism is related to auxin distribution and TRH1 function, and is uncoupled from the inhibition of root elongation in Arabidopsis. J. Exp. Bot. 63, 3777–3788. 10.1093/jxb/ers068 22407650

