# Supplementary figures and images for: CBL-Interacting Protein Kinase OsCIPK18 Regulates the Response of Ammonium Toxicity in Rice Roots
Source: Front Plant Sci. 2022 Apr 29;13:863283. doi: 10.3389/fpls.2022.863283 (PMC9100847; doi:10.3389/fpls.2022.863283)

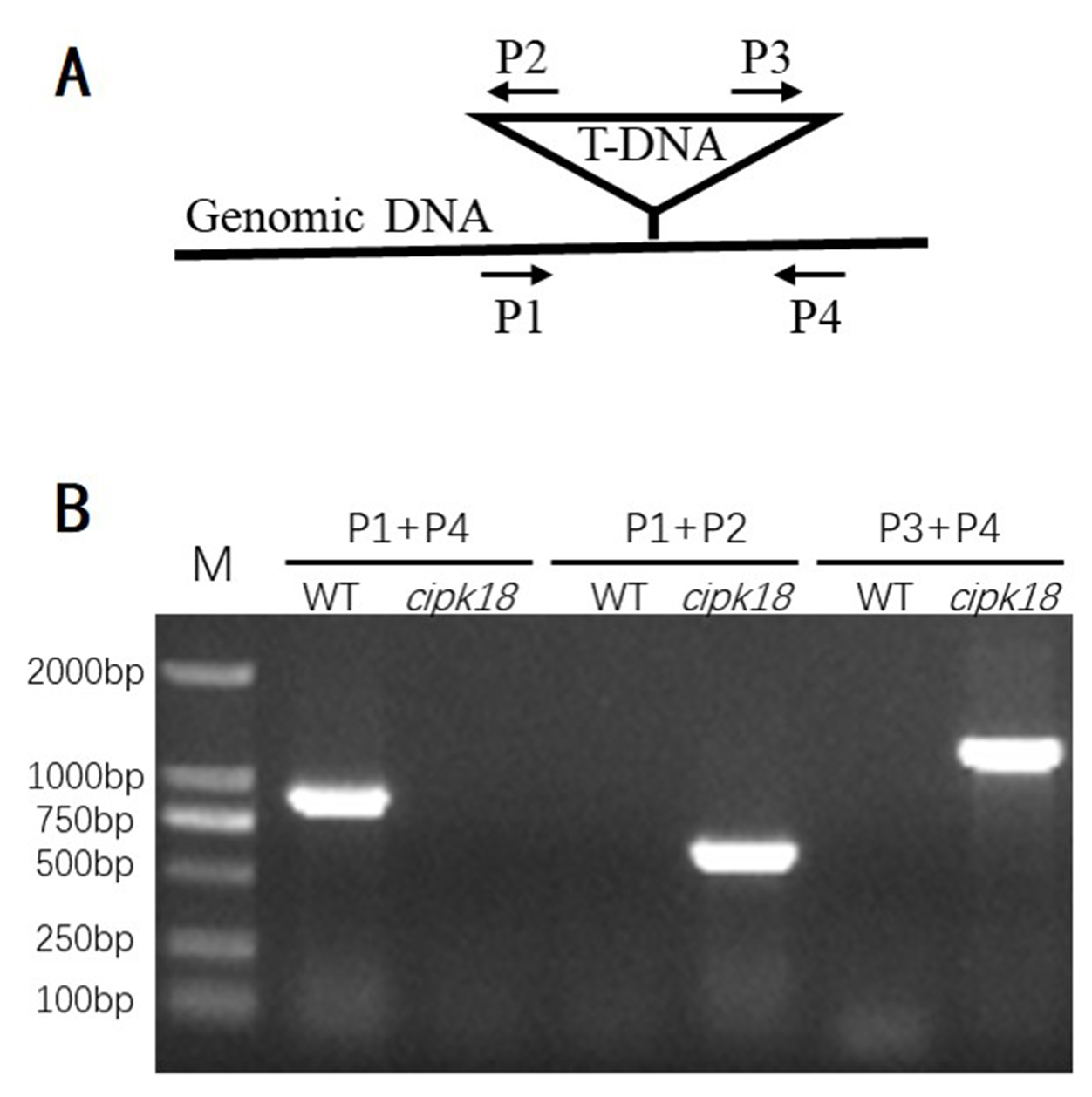

Supplement: Supplementary Figure 1 — Identification of the T-DNA insertion site of cipk18. (A) A schematic map of the T-DNA insertion sites on the genomic regions of OsCIPK18. (B) Detection of the flanking sequence of the T-DNA insertion site in the mutant via PCR/RE assay. [file Image_1.JPEG]

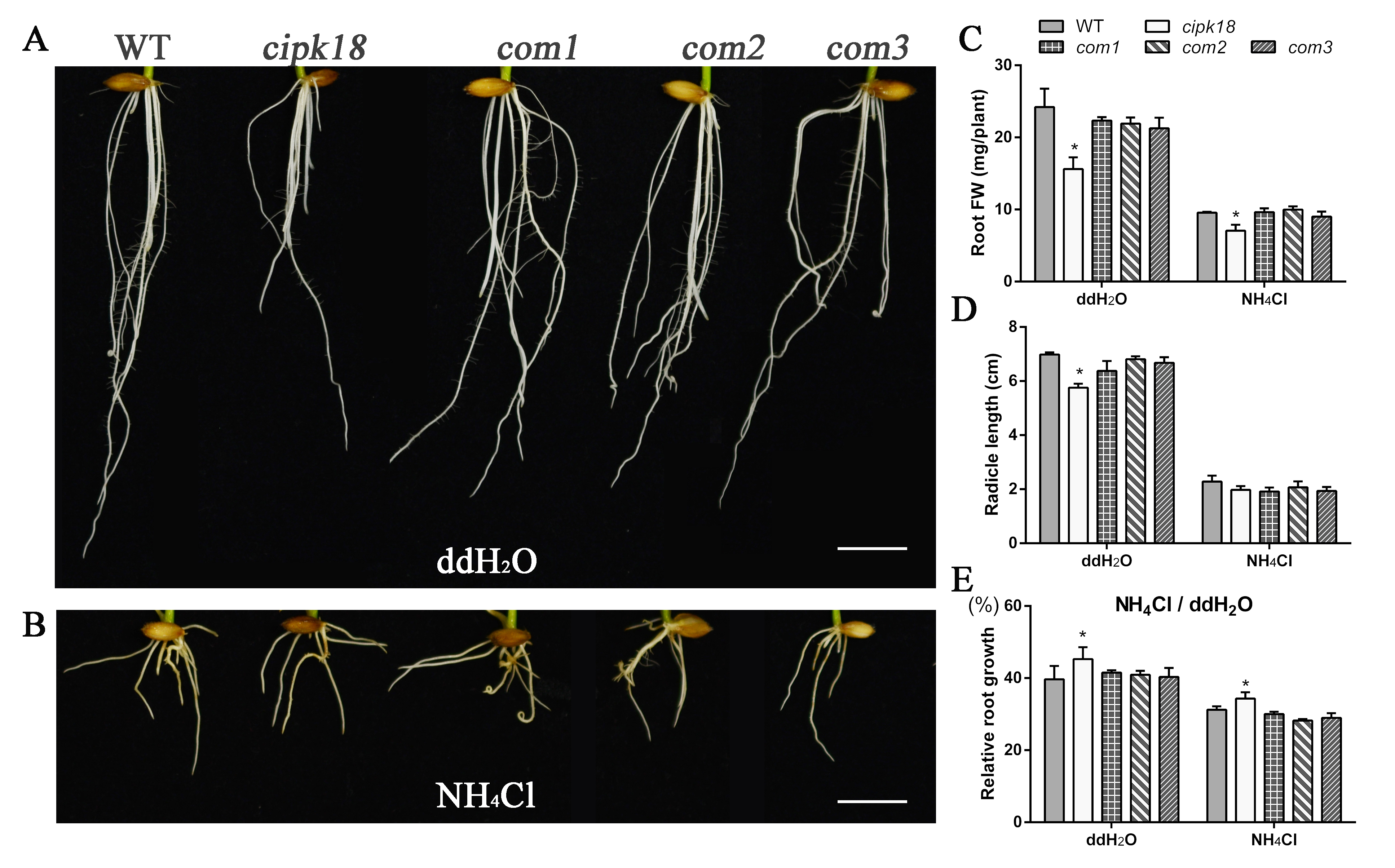

Supplement: Supplementary Figure 2 — The mutant cipk18 shows resistance to excess NH4+. (A–D) Rice seedlings phenotypes (A,B), root FW (C), and radicle length (D) of 5-day-old WT, cipk18, and three complementary materials of cipk18 (com1, com2, and com3) under treatments with ddH2O and 4 mM NH4Cl, respectively. Scale bar = 1 cm. (E) Compared with the control, relative root growth of the five lines under 4 mM NH4Cl. Data are means ± SDs, significant differences using Student's t-test: *p < 0.05. [file Image_2.JPEG]
